# In vitro evaluation of a computer-assisted decision support system for the primary care of polytrauma patients

**DOI:** 10.1007/s00068-023-02295-9

**Published:** 2023-06-08

**Authors:** Christoph Vogel, Juliane Neumann, Lisa Kießling, Gunther Hempel, Thomas Neumuth, Christian Kleber, Georg Osterhoff

**Affiliations:** 1 Department of Orthopaedics, Trauma and Plastic Surgery, University Hospital Leipzig, 04103 Leipzig, Germany; 2 Innovation Center Computer Assisted Surgery (ICCAS), Medical Faculty, University of Leipzig, Leipzig, Germany; 3 Department of Anaesthesiology and Intensive Care, University Hospital Leipzig, Leipzig, Germany

**Keywords:** TraumaFlow, Trauma, Polytrauma, Emergency room, Life support, Workflow management, Decision making, ATLS, Simulation, Safety, Outcome

## Abstract

**Introduction:**

The management of polytraumatized patients is set in a stressful environment with numerous critical decisions in a brief amount of time. Working along a standardised procedure can improve the outcome for these patients and reduce mortality. To help clinical practitioners, we developed “TraumaFlow”, a workflow management system for the primary care of polytrauma patients based on the current treatment guidelines. This study sought to validate the system and investigate its effect on user performance and perceived workload.

**Methods:**

The computer-assisted decision support system was tested in two scenarios in a trauma room of a level 1 trauma centre by 11 final-year medical students and 3 residents. In simulated polytrauma scenarios, the participants functioned as a trauma leader. The first scenario was performed without decision support and the second with support by “TraumaFlow” via tablet. During each scenario, the performance was evaluated in a standardized assessment. After each scenario, the participants answered a questionnaire on workload [NASA Raw Task Load Index (NASA RTLX)].

**Results:**

In total, 14 participants (mean 28 ± 4 years, 43% female) managed 28 scenarios. During the first scenario without computer-assisted support, the participants achieved a mean of 6.6 out of 12 points (SD 1.2, range 5 to 9). With the support of TraumaFlow, the mean performance score was significantly higher with 11.6 out of 12 points (SD 0.5, range 11 to 12, *p* < 0.001). In the 14 scenarios performed without support, there was no run in which no errors were made. In comparison, ten of the 14 scenarios performed with TraumaFlow ran free of relevant errors. The mean improvement in the performance score was 42%. There was a significant decrease in the mean self-reported mental stress level in scenarios with support of TraumaFlow (55, SD 24) as compared to scenarios without support (72, SD 13, *p* = 0.041).

**Conclusion:**

In a simulated environment, computer-assisted decision-making improved the performance of the trauma leader, helped to adhere to clinical guidelines, and reduced stress in a fast-acting environment. In reality, this may improve the treatment outcome for the patient.

## Introduction

In-hospital primary care of polytrauma patients goes along with numerous critical decisions in a brief amount of time and a stressful environment. Under these circumstances, even experienced trauma teams can make errors in treatment [1]. It has been shown that the standardisation of clinical workflows by the application of guidelines and algorithms like ATLS^®^ can reduce the morbidity and mortality of trauma patients [2]. Computer-based decision support systems may assist trauma teams in real time during the primary survey of trauma patients. This could increase accordance with trauma guidelines, reduce the team’s stress levels, and ideally improve the treatment outcome for the patient.

**Fig. 1:**
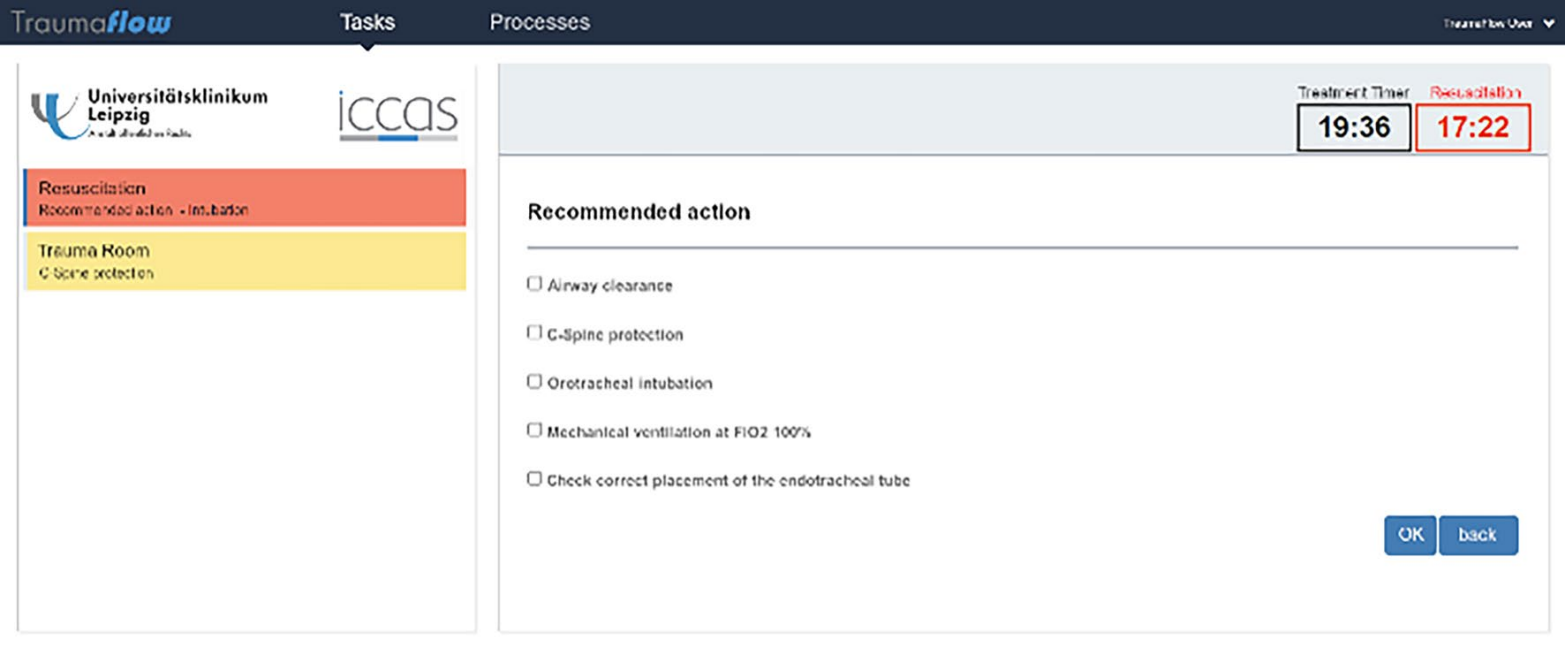
Screenshot of the TraumaFlow user surface

The authors developed “TraumaFlow”, a computer program for the primary care of polytrauma patients (Fig. 1). This workflow management system is based on the German “S3 guideline on the treatment of patients with severe/multiple injuries” [3] and the ATLS^®^ guidelines [4]. TraumaFlow can be used to register, document, and coordinate primary trauma care as well as provide recommendations for the treatment of the patient.

The aim of this study was to validate the system in simulated polytrauma scenarios. Thereby, the overall goal was to investigate the effect of a computer-assisted workflow management system on guideline adherence and subjective workload of trauma leaders during the management of polytrauma patients.

## Methods

### Participants

To measure the effect of TraumaFlow as unaffected as possible by participant expertise, 11 participants with limited experience in treating polytrauma patients were included and as compared to the performance of three senior residents. Inexperienced participants were 11 medical students in their last year. Of these 11 students, eight had experienced ≤ 5 polytrauma managements, while 3 had experienced 6 to 10 cases before the study. The basic principles of ATLS^®^ are part of the curriculum in medical school. Personal data collected on the participants included age, gender, and information on medical training level and experience in the treatment of polytrauma patients. All participants used the same prototype version of TraumaFlow, a computer-based decision support system developed by the authors that is run on a tablet computer.

The protocol of this study was approved by the local ethics committee (reference 118/22-ek).

### Scenarios

**Fig. 2:**
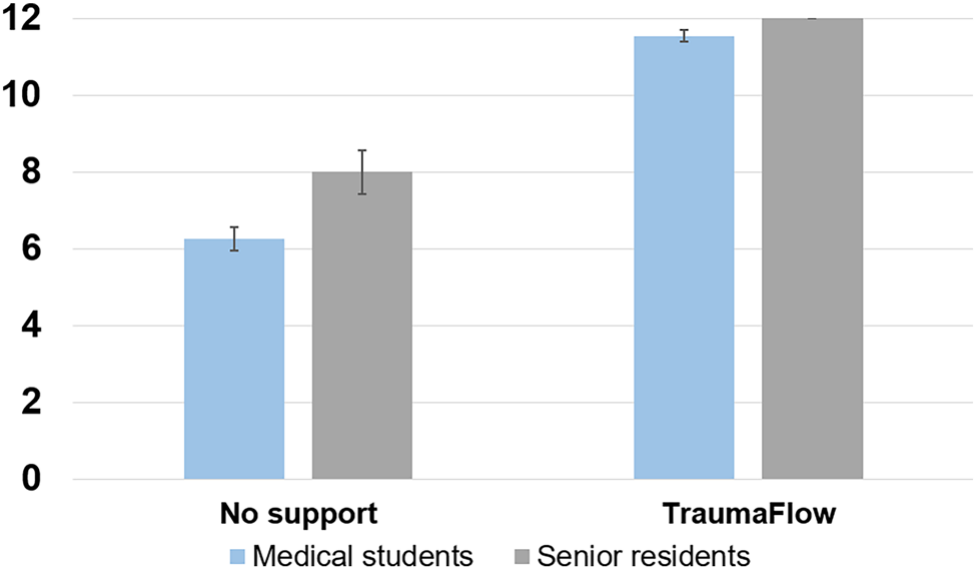
Performance with and without computer-assisted support by the level of expertise of the participants (scale from 0 to maximum 12 points)

In the trauma room of a level 1 trauma centre, two scenarios of polytrauma patients were simulated successively on a simulation manikin (Resusci Anne-Simulator, Laerdal Medical, Stavanger, Norway. Figure 2 together with virtual patient monitoring (Sim.Care EASY 2, deltaplex UG, Hamburg, Germany with TruMonitor-App, TruCorp Ltd., Northern Ireland).

Case A was a 32-year-old male victim of a violent crime who was attacked with blunt weapons. He presents with tension pneumothorax, a relevant bleeding scalp wound, and a splenic injury with a resultant class III haemorrhagic shock. Case B was a 27-year-old man who fell from a scaffold from a height of 6 m. The patient had a severe traumatic brain injury with intracranial haemorrhage, unstable pelvic injury, and free fluid in the abdomen in the Koller pouch and retroperitoneally with resulting class III haemorrhagic shock.

Participants were asked to assume the role of trauma leader and were provided with a nurse and a resident.

The scenario was steered by a certified trauma simulation instructor, and changes in the patient’s condition were reflected via a simulation monitor or verbal instructions.

The first scenario was performed by the participants without support, the second scenario was performed with the assistance of the “TraumaFlow” workflow management system via a tablet. The sequence of cases changed with each participant (A-B, B-A, A-B, …).

### Assessment of performance

During each scenario, a standardized assessment form ranging from 0 to 12 points was used to evaluate the participant on how the patient’s injuries and critical conditions were recognized and whether treatment measures were initiated in accordance with the principles and priorities of ATLS^®^ according to the ATLS^®^ Student Manual 10th edition [4] and the Guideline on the Management of Severly Injured Patients of the German Trauma Society [3]. Before the testing, twelve critical tasks were defined for each scenario, which needed to be completed during the training scenario. One point was given for completing each task resulting in a maximum score of 12 points. Manual skills, e.g., intubation or chest drain insertion, were not assessed.

### Assessment of perceived workload

After each scenario, the participants were asked to complete questionnaires on perceived workload during the simulated cases. For this purpose, the NASA Raw Task Load Index (NASA-RTLX) was utilized [5]. The NASA-RTLX is a multidimensional score with six subscales: mental demand, physical demand, temporal demand, frustration, effort, and performance. After the completion of each scenario, the performed tasks were rated by the participant in the subscales within a 100-points range. In addition, the overall workload is calculated by a combination of all six dimensions.

### Statistical analysis

Statistical analyses were performed using SPSS 27.0 (SPSS Inc., Chicago, IL, USA). The data were summarized as mean with standard deviation (SD). Where applicable, nominal variables crosstabs were associated using Chi-square or Fisher’s exact tests. Student’s t test was used to detect differences in means of normally distributed continuous data. Paired tests were used for comparison of the two scenarios among the same participant. The level of significance was defined as *p* < 0.05.Assuming an effect of at least 2 performance points (SD 2) when using TraumaFlow and aiming for a power of at least 0.80, the minimum sample size was calculated to be *n* = 10.

## Results

In total, 14 participants (mean 28 ± 4 years, 43% female) managed 28 scenarios. Eight of the participants were students in their last year of medical school, three were senior residents.

Four participants had never participated in the treatment of a polytrauma patient in reality, five participants had participated in one to five cases, and five in more than five cases.

Seven participants performed case A without support, followed by case B with support of “TraumaFlow”; the other seven participants performed case B without support, followed by case A with support of “TraumaFlow”.

### Performance

During the first scenario without computer-assisted support, the participants achieved a mean score of 6.6 points (SD 1.2, range, 5 to 9). With the support of TraumaFlow, the mean performance score was significantly higher with 11.6 points (SD 0.5, range 11 to 12, *p* < 0.001). Ten of 14 scenarios with TraumaFlow support were performed without relevant errors, while this was not the case in any of the 14 scenarios without support. The mean improvement in the performance score was 42%.

With the data available, no significant difference in scores could be detected between medical students and the three senior residents, neither without (*p* = 0.225) nor with computer-assisted support (*p* = 1.0, Fig. 2).

### Perceived workload

**Fig. 3:**
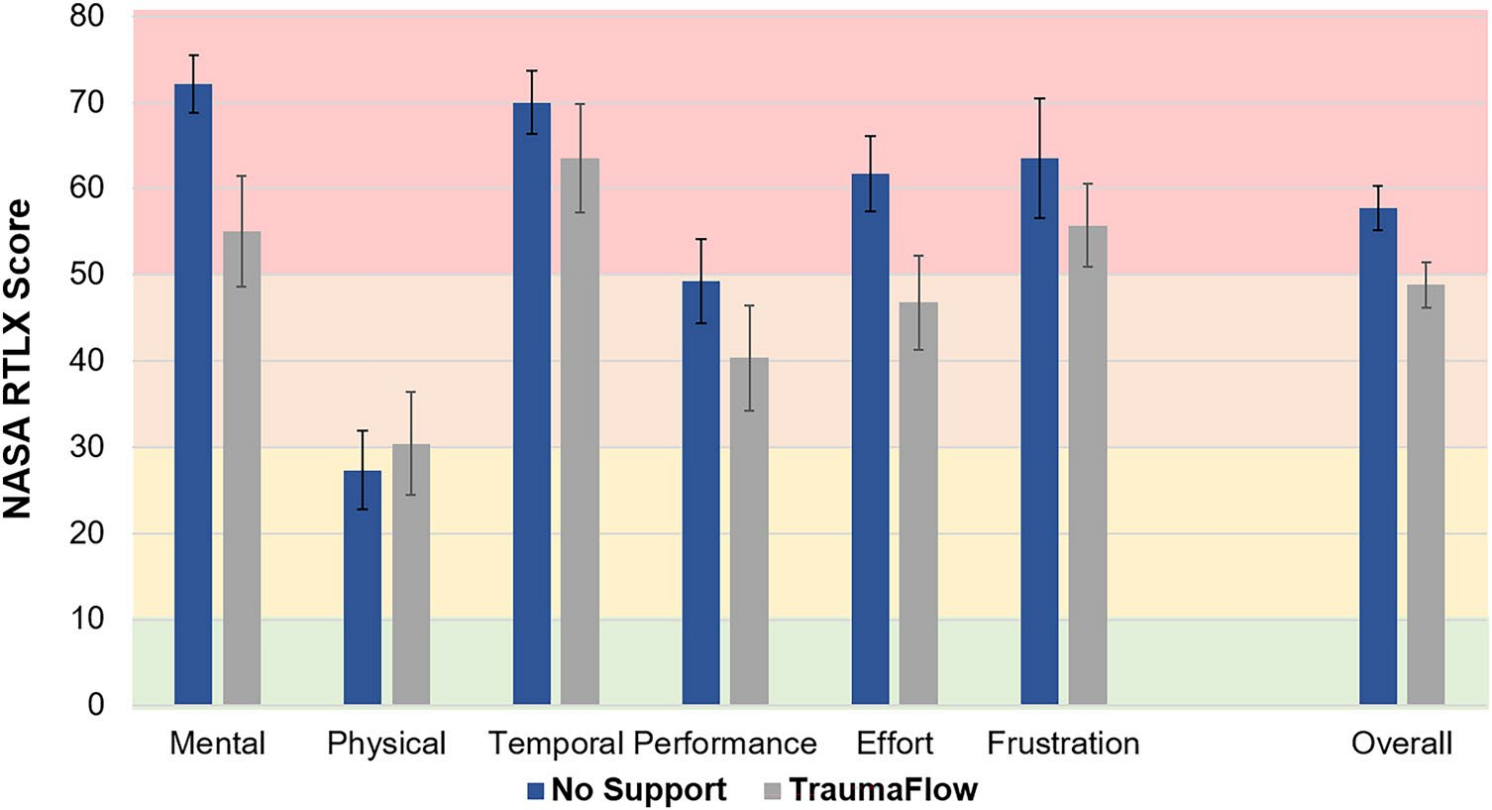
Stress as measured by NASA TXL with and without computer-assisted support

The subjective overall workload was rated as 'high’ by ten participants and ‘somewhat high’ by four participants without decision support (Fig. 3). With the decision-support of TraumaFlow the subjective overall workload was rated as ‘high’ by six participants, ‘somewhat high’ by seven participants and ‘medium by’ one participant. There was no significant difference in the NASA-RTLX overall score between scenarios with (48, SD 14) and without TraumaFlow (57, SD 9, *p* = 0.158). However, there was a significant decrease in the mean self-reported mental stress level in scenarios with support of TraumaFlow (55, SD 24) as compared to scenarios without support (72, SD 13, *p* = 0.041). No differences were found for the NASA-RTLX dimensions of physical stress (*p* = 0.633), temporal stress (*p* = 0.402) and performance (*p* = 0.128), effort (*p* = 0.112), and frustration (*p* = 0.421).

## Discussion

In a simulated in vitro setting, this study sought to investigate the effect of a computer-assisted workflow management system (“TraumaFlow”) on guideline adherence and subjective workload and stress levels of trauma leaders during the management of polytrauma patients. It was found that the support of a computer-assisted workflow management system results in a significant reduction of relevant errors in patient treatment and improves guideline adherence. Furthermore, TraumaFlow was able to significantly reduce self-reported mental stress during the scenarios without increasing the overall workload.

These findings are consistent with the previous studies by Clarke et al. [6], who used a software called TraumaAID, which, unlike Traumaflow, cannot provide context-sensitive information about an individual patient in real time during polytrauma treatment. After training with the programme, TraumaAID was able to reduce the amount of preventable clinically relevant mistakes by 98%. It has been shown that the clinical implementation of algorithms and a higher grade of standardisation leads to better outcomes [2, 7]. A digital and easily accessible guide that improves guideline adherence like TraumaFlow, hence, is a leap in this direction as has been discussed previously [8].

In contrast to the previous studies, we looked at the impact of a computer-based decision aid on stress levels, in addition to the accuracy of treatment. Another difference is that TraumaFlow was configured according to the current guidelines and the established ATLS® standard according to the 10th student manual.

Although there seem to be significant benefits to using a workflow management system in the treatment of trauma patients, it is unlikely an instrument that can immediately be installed in all hospitals. The advantages of TraumaFlow depend on the correct and complete usage of the system. Some participants did not like the increased dependency on external devices compared to unsupported scenarios. The manual input of patient and treatment parameters led to frustration and stress in some cases.

Hence, it would be essential to properly train users before the implementation of such a system in order to reduce further stress and maximize the quality of treatment. Automated collection of parameters from the monitoring devices or cameras would be another step towards a better workflow assistance when using TraumaFlow. Beyond this, TraumaFlow can only work correctly with the necessary digital infrastructure like tablets and wireless networks. The introduction of a workflow system initially produces more costs for the hospital without being refinanced through health insurance. On the other hand, in the long run, both hospitals and the healthcare system could benefit from the reduction of errors and thus reduction of complications and saving of resources.

A chaotic workplace and stress have been linked to errors in treatment and a poorer quality of care with increased costs [9]. Even in this simulated and protected environment, the participants of this study ranked their stress level between “high” and “somewhat high”. We were able to show that the computer-assisted decision support system could reduce stress significantly and it may therefore contribute to patient safety.

The two case scenarios that were used in this study included typical life-threatening situations that usually can be addressed when adhering to the referenced guidelines (tension pneumothorax, open bleeding wound, abdominal haemorrhage, unstable pelvic injury, haemorrhagic shock). Polytrauma patients are a very heterogeneous group of patients that are not comprehensively described by these two scenarios. This is a limitation of this study. However, this study was not designed to prove that a computer-assisted decision support system improves patient outcome. The aim of the study was to show that such a system is able to improve guideline adherence of inexperienced users in stressful situations like polytrauma management.

We chose mainly unexperienced participants in order to see the effect of TraumaFlow unbiased by experience. It may be that the beneficial effect for experienced trauma leaders does not outweigh the additional workload caused by using the application as they are already used to trauma room management and have their own routines, while working with TraumaFlow is additional work for them. It should be noted, however, that such a technical support can be a useful support especially for centres with a low number of cases and less experience in the management of severely injured patients. The reality in many hospitals with a low case volume is that the trauma leader will likely not be experienced in the management of trauma patients or be a qualified ATLS® provider. Systems like TraumaFlow can help to adhere to guidelines and standards the user may not be trained in.

The current TraumaFlow prototype is not a medical device, though, and this study was just a proof of concept that such a system could improve polytrauma management. Before TraumaFlow can be used in real patient treatment, its effect and usability in real-life situations must be investigated in future studies to consolidate its validity.

The next step would be to integrate artificial intelligence applications into such systems. Various studies have shown that AI-assisted decision-making systems have the potential to improve outcome in the treatment of trauma patients [8]. For further implementation in patient care, however, technological barriers need to be overcome and more data of the very heterogeneous trauma patient cohort needs to be collected.

## Conclusion

In a simulated environment,** c**omputer-assisted decision-making improved the performance of the trauma leader, helped to adhere to clinical guidelines, and reduced stress in a fast-acting environment.

## Data Availability

Raw data can be provided on request from the corresponding author.
